# Longitudinal Diffusion Tensor Imaging Resembles Patterns of Pathology Progression in Behavioral Variant Frontotemporal Dementia (bvFTD)

**DOI:** 10.3389/fnagi.2018.00047

**Published:** 2018-03-06

**Authors:** Jan Kassubek, Hans-Peter Müller, Kelly Del Tredici, Michael Hornberger, Matthias L. Schroeter, Karsten Müller, Sarah Anderl-Straub, Ingo Uttner, Murray Grossman, Heiko Braak, John R. Hodges, Olivier Piguet, Markus Otto, Albert C. Ludolph

**Affiliations:** ^1^Department of Neurology, University of Ulm, Ulm, Germany; ^2^Clinical Neuroanatomy, Department of Neurology, University of Ulm, Ulm, Germany; ^3^Department of Clinical Neurosciences, University of Cambridge, Cambridge, United Kingdom; ^4^Max Planck Institute for Human Cognitive and Brain Sciences & Clinic for Cognitive Neurology, University Hospital, Leipzig, Germany; ^5^Department of Neurology, Perelman School of Medicine, University of Pennsylvania, Philadelphia, PA, United States; ^6^School of Medical Sciences, University of New South Wales, Sydney, NSW, Australia; ^7^ARC Centre of Excellence in Cognition and its Disorders, Sydney, NSW, Australia

**Keywords:** frontotemporal lobar degeneration, diffusion tensor imaging, fractional anisotropy, neuropathology, staging

## Abstract

**Objective:** Recently, the characteristic longitudinal distribution pattern of the underlying phosphorylated TDP-43 (pTDP-43) pathology in the behavioral variant of frontotemporal dementia (bvFTD) excluding Pick's disease (PiD) across specific brain regions was described. The aim of the present study was to investigate whether *in vivo* investigations of bvFTD patients by use of diffusion tensor imaging (DTI) were consistent with these proposed patterns of progression.

**Methods:** Sixty-two bvFTD patients and 47 controls underwent DTI in a multicenter study design. Of these, 49 bvFTD patients and 34 controls had a follow-up scan after ~12 months. Cross-sectional and longitudinal alterations were assessed by a two-fold analysis, i.e., voxelwise comparison of fractional anisotropy (FA) maps and a tract of interest-based (TOI) approach, which identifies tract structures that could be assigned to brain regions associated with disease progression.

**Results:** Whole brain-based spatial statistics showed white matter alterations predominantly in the frontal lobes cross-sectionally and longitudinally. The TOIs of bvFTD neuroimaging stages 1 and 2 (uncinate fascicle—bvFTD pattern I; corticostriatal pathway—bvFTD pattern II) showed highly significant differences between bvFTD patients and controls. The corticospinal tract-associated TOI (bvFTD pattern III) did not differ between groups, whereas the differences in the optic radiation (bvFTD pattern IV) reached significance. The findings in the corticospinal tract were due to a “dichotomous” behavior of FA changes there.

**Conclusion:** Longitudinal TOI analysis demonstrated a pattern of white matter pathways alterations consistent with patterns of pTDP-43 pathology.

## Introduction

Frontotemporal lobar degeneration (FTLD) is the second most frequent cause of dementia and, in individuals under 65 years of age, its prevalence is similar to that of Alzheimer's disease (Rosso et al., 2013). The neuropathological hallmarks of a large proportion of FTLD cases are neuronal cytoplasmic inclusions and dystrophic neurites that are immunoreactive for ubiquitin, p62, and the phosphorylated 43-kDa TAR DNA-binding protein (pTDP-43), which is also the major disease protein in amyotrophic lateral sclerosis (Neumann et al., 2006). The progressive accumulation of inclusions formed by disease-specific protein aggregates is typical of many neurodegenerative diseases, including sporadic Alzheimer's disease and sporadic Parkinson's disease (Jucker and Walker, 2013; Goedert, 2015; Walker and Jucker, 2015). Importantly, in postmortem studies, many of these disorders reveal a characteristic distribution pattern of their underlying pathology across specific brain regions with disease progression, as was recently proposed for pTDP-43 proteinopathies (Brettschneider et al., 2013, 2014; Josephs et al., 2014). These findings may permit the categorization of longitudinal disease progression into disease stages in a clinical setting (bvFTD stages). Although challenging, the transfer of neuropathological findings into a clinical setting could make individual diagnostic procedures possible and encourage its use as a potential surrogate marker of disease to achieve substantial cost-savings and increase the feasibility of clinical trials (McMillan et al., 2014). Recently, we demonstrated that progressive regional accumulation of pTDP-43 inclusions in ALS reflects sequential involvement of white matter fiber tracts of the central nervous system (Kassubek et al., 2014, 2017; Müller et al., 2016). While no direct neuroimaging marker for pTDP-43 exists at present, several earlier neuropathological studies have emphasized the close correlation between the severity of pTDP-43 aggregation and neuro-axonal loss (Geser et al., 2008, 2009). Behavioral variant frontotemporal dementia (bvFTD), the main FTLD subtype, is characterized by progressive decline of executive function and inappropriate social conduct (Rascovsky et al., 2011). Four neuropathological patterns of pTDP-43 pathology were recently identified in a cohort of 39 autopsy cases excluding Pick's disease (PiD) (Brettschneider et al., 2014). Consistent with the hypothesis of sequential progression and propagation along axonal pathways, the four regional distribution patterns of pTDP-43 pathology included: (I) basal and anterior portions of the prefrontal neocortex (orbital gyri, gyrus rectus, inferior frontal gyrus), (II) prefrontal neocortical areas (middle frontal gyrus, insular cortex, and anterior cingulate gyrus), anteromedial temporal areas (transentorhinal region), the superior and middle temporal gyri, striatum, red nucleus, medial and lateral portions of the thalamus, and precerebellar nuclei of the brainstem, (III) the agranular motor neocortex, parietal neocortical areas (sensory cortex, angular gyrus), bulbar somatomotor neurons, and (IV) the occipital neocortex. In the current study, fiber tracking with a tract of interest (TOI)-based approach was applied to a cohort of bvFTD patients and control individuals both cross-sectionally and longitudinally to analyze involved major white matter pathways and to test the hypothesis that a pattern corresponding to those areas seen at autopsy during staging could be confirmed *in vivo*. The aim of this study was two-fold: (1) based on longitudinal DTI data, microstructural longitudinal alterations should be detectable in the course of bvFTD. (2) Based on previous neuropathological findings and on the *in vivo* microstructural cross-sectional and longitudinal alteration and progression patterns, (2) it should be possible to propose a staging protocol using a tract-based DTI analysis that allows for an individual staging analysis for bvFTD patients.

## Methods

### Subjects and MRI acquisition

Sixty-two patients with a clinical diagnosis of bvFTD according to established diagnostic criteria (Rascovsky et al., 2011) as made by specialized board-certified neurologists were included in this study, together with 49 controls. Data acquisition was performed at three study sites (Ulm and Leipzig, Germany; Sydney, Australia). The study was approved by the local ethics committees (Ethics Committee of the University of Ulm (39/11); Ethics Committee of the University of Leipzig (137-11-18042011); Human Ethics Committees of South Eastern Sydney and Illawarra Area Health Service and the University of New South Wales), and written informed consent was obtained from each participant or the primary caregiver in accordance with the Declaration of Helsinki. Demographic data are shown in Table 1. BvFTD was diagnosed after clinical examination, neuropsychological testing, and MRI findings, according to the criteria of the Lund-Manchester Group (Neary et al., 1988; The Lund and Manchester groups, 1994) and the Work Group on Frontotemporal Dementia and Pick's Disease (McKhann et al., 2001). Patients with bvFTD displayed progressive behavioral abnormalities with an early loss of personal and social insight and, in MRI, symmetrical or asymmetrical atrophy in the anterior temporal lobes, prefrontal lobes, or both.

**Table 1:** Demographic parameters for bvFTD patients (*N* = 62) and controls (*N* = 47).

	***N***	***N* (follow-up)**	**Age/years**	**Gender (m/f)**	**Disease duration/years**	**MMSE**
**Ulm, Germany**						
bvFTD	18	10	65 ± 11	7/11	3 ± 2	25 ± 5
Controls	15	6	63 ± 12	9/6	n.a.	n.a.
**Leipzig, Germany**						
bvFTD	10	5	62 ± 13	5/5	6 ± 8	24 ± 4
Controls	7	3	61 ± 6	3/4	n.a.	n.a.
**Sydney, Australia**						
bvFTD	34	34	62 ± 8	23/11	5 ± 3	n.av.
Controls	25	25	69 ± 7	11/14	n.a.	n.a.
**Sum**						
bvFTD	62	49	63 ± 10	35/27	5 ± 4	24 ± 5
Controls	47	34	66 ± 9	23/24	n.a.	n.a.
P			> 0.05	> 0.05		

*Values are given in mean (± standard deviation). MMSE, Mini-Mental-State-Examination (MMSE) (Folstein et al., 1975), n.a., not applicable; n.av. not available. p was calculated by Mann-Whitney test*.

The bvFTD patients and controls from all centers underwent MR scanning on 3.0T scanners (Sydney: 3.0T Philips; Leipzig: Verio, Siemens Medical; Ulm: Allegra, Siemens Medical). Accordingly, DTI data were acquired using three different protocols. The Ulm DTI protocol consisted of 31 gradient directions (GD), including one b = 0 GD (80 slices, 112 × 128 pixels; slice thickness was 2.0 mm, in-plane pixel size was 2.0 × 2.0 mm). The echo time (TE) and repetition time (TR) were 88 and 11100 ms; b was 1000 s/mm^2^. The Leipzig DTI protocol consisted of 67 GD, including 7 b = 0 GD (80 slices, 128 × 128 pixels; slice thickness was 1.7 mm, in-plane pixel size was 1.7 × 1.7 mm); TE and TR were 100 and 15200 ms; b was 1000 s/mm^2^. The Sydney DTI protocol consisted of 34 GD, including one b = 0 GD (55 slices, 96 × 96 pixels; slice thickness was 2.5 mm, in-plane pixel size was 2.5 × 2.5 mm); TE and TR were 68 ms and 8400 ms; b was 500 s/mm^2^.

During a follow-up scan with a time-interval of 12 months (range 10–41 months), 49 of the 62 bvFTD patients received a DTI scan that allowed further analysis; 34 of the 47 controls received a follow-up scan with a time-interval of 20 months (range 12 to 59 months). The remaining 13 patients were not available for a second MRI investigation due to inconveniences caused by long travel.

### Standardized data pre-processing

The DTI analysis software *Tensor Imaging and Fiber Tracking* (TIFT) (Müller et al., 2007a, 2013) was used for pre-/post-processing and statistical analysis.

#### Data processing I: MNI normalization

After motion correction of individual DTI data sets, in a first step, baseline DTI and follow-up DTI data were aligned by fitting of the (b = 0)-volumes (intensity difference minimization); here, halfway linear registration matrices (Menke et al., 2014) were applied to avoid a bias of the baseline data. Consequently, the following stereotaxic MNI transformation of baseline and follow-up data were performed with the identical parameters.

**Figure 1:**
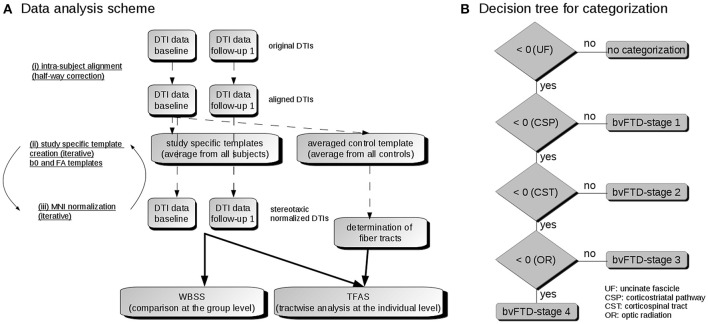
**(A)** Data analysis scheme. (i) To obtain a common coordinate frame, baseline and follow-up DTI data were aligned by half-way correction. (ii) After landmark normalization, study-specific b0 and FA templates were created. (iii) DTI data of all visits were stereotaxically normalized in the Montreal Neurological Institute (MNI) coordinate frame in an iterative process. Then, the voxelwise statistical comparison between the patients and the control group was performed by whole brain based spatial statistics (WBSS). After averaging controls' data sets, fiber tracts were calculated from this averaged data set. Finally, tractwise fractional anisotropy statistics (TFAS) was applied. **(B)** Decision algorithm for categorization. From all bvFTD patients, those were further analyzed who had z-transformed TOI-FA values < 0, i.e., TOI-FA values below the FA-threshold defined for the uncinate fascicle (bvFTD-stage 1). Of this group, those were defined for bvFTD-stage 1 who had z-transformed TOI-FA-values > 0 in the corticostriatal pathway, and those were defined for bvFTD-stage 2 who had z-transformed TOI-FA-values > 0 in the corticospinal tract. The remaining individuals were categorized into bvFTD-stages 3 or 4, depending on whether their z-transformed TOI-FA-values in the optic radiation were > 0 or < 0.

Spatial normalization to the Montreal Neurological Institute (MNI) stereotaxic standard space was performed (Brett et al., 2002; Figure 1A): The normalization process was iterative, using a study-specific (b = 0)-template and additionally a fractional anisotropy (FA)-template at each iteration step, since an FA-template has more contrast in comparison to (b = 0)-images (Smith et al., 2006). In the first iteration step, a normalization by a linear transformation according to manually set landmarks leads to a first study-specific (b = 0)-template and to an FA-template by arithmetically averaging the (b = 0)-volumes and FA maps, respectively. To optimize the normalization matrices, improved templates could be created in a second iteration step from the normalized DTI data by a non-linear MNI normalization of the DTI data sets (non-linear minimization of the mismatch between regional intensities of the FA-map to be fitted and of the FA-template according to the squared differences). The correlation between individual FA-maps and the FA-template is > 0.7 after two iterations so that the iterative process was stopped after two iterations (Müller and Kassubek, 2013).

After each iteration step, a systematic between-center adjustment was performed (see section “Data processing V”) prior to creation of the study specific templates. Directional information during the normalization process was preserved according to techniques described by Alexander et al. (2001) (Müller et al., 2009).

Finally, a Gaussian smoothing filter of 8 mm full width at the half maximum (FWHM) was applied to the individual normalized FA-maps. The filter size of 8 mm (which is about 2–3 times the recording voxel size, depending on the protocol) provides a good balance between sensitivity and specificity (Müller et al., 2013).

#### Data processing II: definition of tract structures

To apply group-based fiber tracking (FT) algorithms (Müller et al., 2009), an averaged DTI data set was calculated from 25 control data sets with the identical DTI protocol by arithmetic averaging of the MNI transformed data. For each voxel position, Eigenvectors and Eigenvalues were calculated that represented the average of all controls' data sets. For this averaged DTI data set, only controls' data sets were used because the reconstruction of the pathways could be biased if voxels with pathology-induced alterations would contribute to the averaged data set. Directional information of each data set was preserved during the normalization process and was incorporated in the template creation (Müller et al., 2009).

This averaged DTI data set from controls was then used to identify pathways for defined brain structures according to the previously proposed neuropathological patterns (Brettschneider et al., 2014) with a seed-to-target approach (Kassubek et al., 2014, 2017). For this purpose, regions of interest (ROIs) were defined for the seed and target regions. For fiber tracking, only voxels with an FA value above a threshold of 0.2 were considered. All fiber tracts (FTs) originating in the seed ROI and ending in the target ROI of the respective pathway define the corresponding TOI. For the fiber tracking technique, a modified deterministic streamline tracking approach was used that takes the directional information of neighbored FTs into account (Müller et al., 2009).

The specific pathogenic mechanisms underlying bvFTLD are still largely unknown; however, studies of anatomical connectivity provide evidence for white matter impairment in bvFTD (Mahoney et al., 2014, 2015; Tovar-Moll et al., 2014). For this reason, it appeared conceptually reasonable to choose, tracts that might reflect the hypothetical “progression” of the pathology along anatomical pathways (axons) in relationship to relevant regions of the brain:

Pattern 1: TDP-43 pathology develops in projection neurons of basal and anterior portions of the prefrontal neocortex (orbital gyri, gyrus rectus, and inferior frontal gyrus). We opted for the uncinate fascicle, in part, based on the previous findings there in bvFTD by other groups (Mahoney et al., 2014, 2015; Tovar-Moll et al., 2014).

Pattern 2: This pattern is marked by the development of pathology in the middle frontal gyrus, insular cortex, anterior cingulate gyrus, the superior and middle temporal gyri, ventral striatum (accumbens nucleus), the parvocellular portion of the red nucleus, in medial and lateral portions of the thalamus, the inferior olivary complex, but also in anteromedial portions of the temporal lobe: transentorhinal and entorhinal regions), including the hippocampal formation (dentate fascia, Ammon's horn sectors 1-2).

Pattern 3: The chief structures affected are the agranular motor cortex and functionally linked motor neurons in the brainstem and spinal cord ventral horn. Thus, the corticospinal tract (CST) was the logical tract of choice.

Pattern 4: The greatest burden of TDP-43 pathology occurred in the occipital neocortex (visual cortex, Brodmann 17, 18) in addition to involvement of all of the regions affected in the previous three patterns. It should be pointed out that the visual cortex either was not examined in detail or at all in early (pre-TDP-43) post-mortem studies of frontotemporal degenerative disorders (e.g., see Mann et al., 1993; The Lund and Manchester groups, 1994; Giannakopoulos et al., 1995). The optic radiation was considered a suitable choice to represent this pattern.

**Figure 2:**
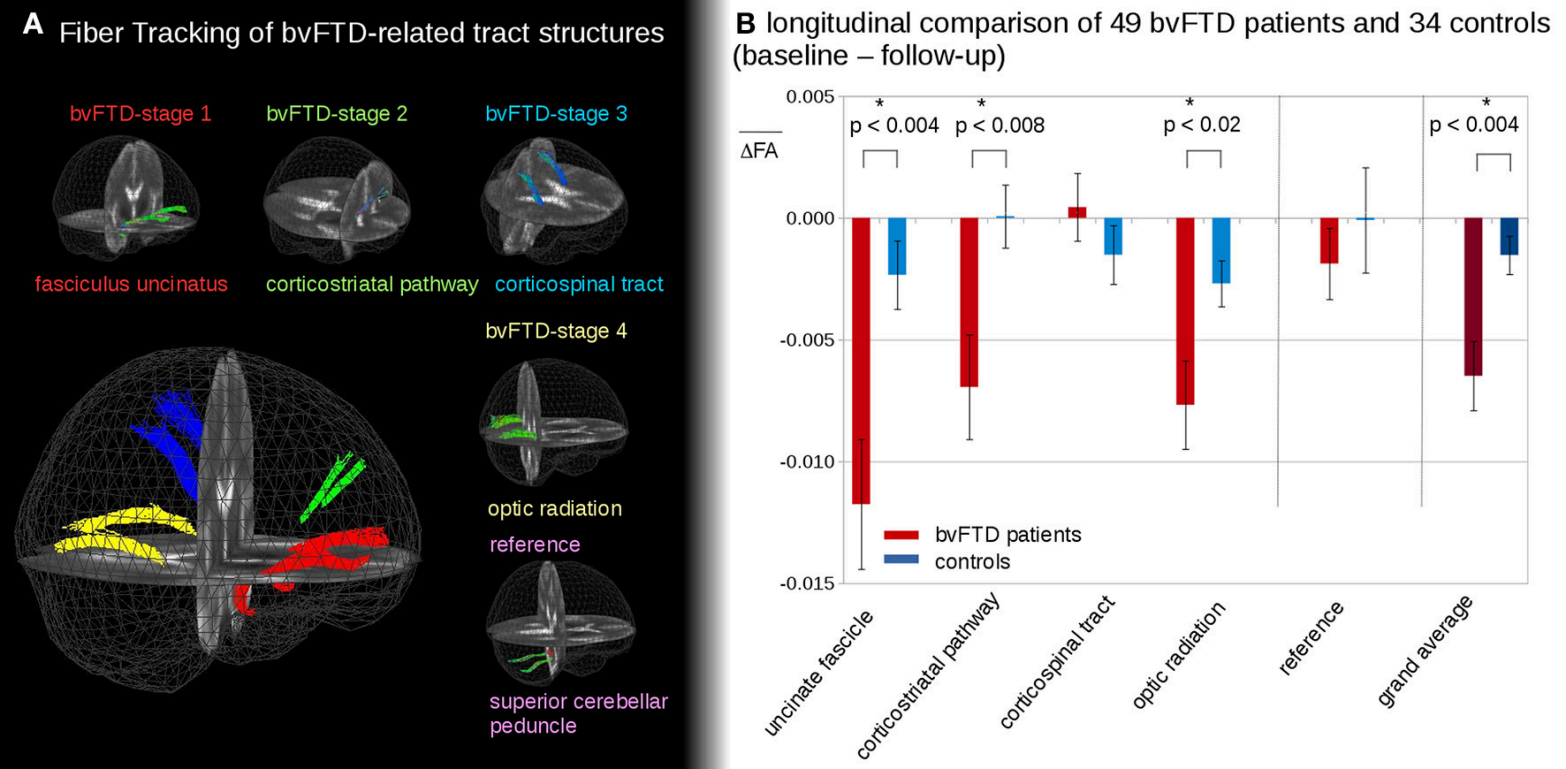
**(A)** 3D-images of tracts of interest. 3D-images of tracts of interest: fasciculus uncinatus, corresponding to bvFTD stage 1; corticostriatal pathway, corresponding to bvFTD stage 2; corticospinal tract (CST) corresponding to bvFTD stage 3, optic radiation, corresponding to bvFTD stage 4; superior cerebellar peduncle as reference path. **(B)** Longitudinal FA differences in stage-related tract systems. Longitudinal averaged FA differences (baseline—follow-up) in tracts at the group level—*significance.

Figure 2 shows the representative TOIs for the definition of the four bvFTD patterns: (I) the frontal part of the uncinate fascicle, (II) the corticostriatal pathway, (III) the corticospinal tract (CST), and (IV) the optic radiation (Brettschneider et al., 2014). Additionally, the superior cerebellar peduncle was defined as a reference path where no involvement in bvFTD-associated neurodegeneration could be anticipated. TOI coordinates are summarized in Table 2.

**Table 2:** Tract-of-interest (TOI) based fiber tracking (FT).

**Tract**		**Seed ROI**	**Target ROI**
Fasciculus uncinatus	bvFTD-stage 1	±39/-1/-23	±14/48/-5
Corticostriatal path	bvFTD-stage 2	±23/22/12	±13/38/36
Corticospinal tract	bvFTD-stage 3	±27/-18/18	±19/-40/58
Optic radiation	bvFTD-stage 4	±45/-40/-3	±26/-82/2
Superior cerebellar peduncle	reference	0/-29/-25	±27/-67/-42

*Montreal Neurological Institute (MNI) coordinates for seed and target ROIs for the respective FTs for the TOI approach. ROI sizes were 10 mm for all seed ROIs and 20 mm for all target ROIs*.

#### Data processing III: tract-wise fractional anisotropy statistics

The technique of tract-wise fractional anisotropy statistics (TFAS) (Müller et al., 2007b) was applied to quantify the tractography results by use of the TOI. The FA values underlying the specific tracts were arithmetically averaged for each stereotaxically normalized DTI data set of each subject. The TOIs that were defined for the four bvFTD patterns, each being neuropathologically defined as becoming involved in a given sequence (Brettschneider et al., 2014), build the basis for “bvFTD-stage”-related results, i.e., FA values underlying the bihemispheric uncinate fascicle were averaged to “bvFTD-stage 1”-related results, and an analogous procedure was applied to the TOIs corresponding to the other three bvFTD-patterns.

### Data analysis of longitudinal differences

#### Data processing IV: comparison of longitudinal alterations at the group level

The FA differences between follow-ups and the baseline in the TOIs of bvFTD patients and controls were directly calculated from MNI-normalized data with prior alignment of baseline and follow-up DTI data (halfway linear registration, Menke et al., 2014). With the assumption that the alteration process is linear, the differences in FA-values were then linearly corrected to the identical time interval of 12 months prior to the comparison at the group level (Kassubek et al., 2017). Differences in FA-values were linearly normalized for each subject (bvFTD patients and controls) to an identical time-interval prior to statistical comparison.

(1) $$\Delta FA=(FA(t_{1})-FA(t_{2}))/{\left(t_{1}-t_{2}\right)}^{*}1d$$

*t*_1_ and *t*_2_ are the timepoints of baseline and follow-up scans.

In this manner, variable follow-up durations were compensated for, especially the (at average) longer follow-up durations of controls. Note that these differences include the normal aging within the time interval of 12 months.

### Cross-sectional data analysis at the group level

#### Data processing V: whole brain-based spatial statistics

Different factors may contribute to the variability of DTI data of bvFTD patients and control subjects. The analysis of group FA differences between controls on systematic between-center differences was used to adjust for these differences in order to make pooling across centers feasible. First, the covariates age and voxel size were regressed out in FA maps of controls, and a corrected FA-map set consisting of FA maps of all controls was calculated. In a second step, a comparison of FA maps of controls samples was performed between centers, and 3-D linear correction matrices were calculated (Rosskopf et al., 2015). Finally, the covariates age and voxel size were regressed out, and the 3-D linear correction matrices were applied to the FA maps of all controls and bvFTD patients from each center to obtain center-harmonized FA maps. This procedure has already been successfully applied to a multicenter data analysis from eight sites (Müller et al., 2016).

WBSS between the patient group and the controls was performed in protocol-harmonized and age-corrected FA maps by Student's *t*-test (Müller et al., 2016). FA values below 0.2 were not considered for calculation (cortical gray matter shows FA values up to 0.2) because this is the most reliable method to distinguish between white and gray matter FA values (Kunimatsu et al., 2004; Müller et al., 2012). The next steps were the correction for multiple comparisons using the false-discovery-rate (FDR) algorithm (Genovese et al., 2002) at *p* < 0.05 and a clustering procedure for further reduction of type I and type II errors with a threshold cluster size of 256 voxels (Müller et al., 2013).

### bvFTD staging

#### Data processing VI: bvFTD staging

Single subject results for each of the pathways was performed by TFAS. For each DTI data set (of each subject), an averaged FA value for each TOI was obtained. Following the previously reported analysis procedure (Kassubek et al., 2014), optimum group separation (optimization of sensitivity and specificity for group separation in the primary affected tract structure, i.e., the frontal part of the uncinate fascicle), was obtained at an FA-threshold of μ-0.47σ and was chosen to categorize the disease stage between bvFTD subjects and controls (Kassubek et al., 2014). By application of analogous thresholds (μ-0.47σ) for TFAS to the remaining tract systems, this categorization into the staging scheme (“bvFTD stage 1” to “bvFTD stage 4”) was performed after z-transformation of FA results to the respective FA threshold (defined separately for each TOI structure; Figure 1B): bvFTD patients who had z-transformed TOI-FA values < 0 in the TOI of the respective bvFTD stage were further analyzed for the following bvFTD stages, i.e., TOI-FA values below the FA-threshold defined for the frontal part of the uncinate fascicle (related to bvFTD stage 1). Of this group, those were defined for bvFTD stage 1 who had z-transformed TOI-FA-values > 0 in the corticostriatal pathway (related to bvFTD stage 2), and those were defined for bvFTD stage 2 who had z-transformed TOI-FA-values > 0 in the corticospinal tract (related to bvFTD stage 3). The remaining were categorized into bvFTD stages 3 or 4, depending if their z-transformed TOI-FA-values in the optic radiation (related to bvFTD stage 4) were > 0 or < 0.

### Statistics

#### Regression calculations

From the controls' FA maps for every voxel, a regression line of FA values depending on the age of the subjects was calculated:

(2) $$FA_{i}=a_{i}^{*}Age+b_{i}$$

*i* denotes voxel *i*, and the 3D-matrix of *a*_*i*_ was used to calculate the age-normed FA_i_ values for voxel *i* in each subject's FA map.

#### Scanner corrections

Differences of FA maps derived from different DTI protocols were assumed to be approximated by a polynomial approach (Rosskopf et al., 2015)

(3) $$y=a_{0}+a_{1}x+a_{2}x^{2}+\ldots +a_{n}x^{n}$$

where *y*_*i*_ and *x*_*i*_ were the voxel-FA values of the different samples at MNI position *i*; *a*_*n*_ were the respective coefficients.

The polynomial approach offers the possibility of defining the differences between FA maps of the different samples (e.g., from two scan protocols A and B) as

(4) $$\left(\text{i})\text{ linear shift}:\;{\text{a}}_{0}\neq 0;{\text{a}}_{1}\;\text{=}\;1;{\text{a}}_{\text{n}}\;\text{=}\;\text{0 }(\text{n}\;>\;1\right)$$

(5) $$\begin{array}{l} \left(\text{ii})\text{ linear regression}:\;{\text{a}}_{0},{\text{a}}_{1}\neq \text{0};{\text{a}}_{\text{n}}\;\text{=}\;\text{0 }(\text{n}\;>\;1\right)\\ (\text{iii})\text{ higher order differences}. \end{array}$$

That way, a linear shift 3D correction matrix could be calculated where each voxel position represents the difference of averaged FA values of controls (protocol A) and averaged FA values of controls (protocol B). A linear regression 3D correction matrix or higher order differences could not be calculated due to a restricted number of control subjects. NB: The linear shift approximation *a*_0_ ≠ *0; a*_1_ = *1; a*_*n*_ = *0 (n* > *1)* reveals similar results as *a*_0_ = *0; a*_1_ ≠ *0; a*_*n*_ = *0 (n* > *1)*.

#### Correlations

For correlation calculations, Pearson's correlation coefficient *R* was used.

## Results

### Differences in longitudinal TFAS at the group level

In the follow-up measurements of the 49 bvFTD patients and the 34 controls, FA differences in the tract systems showed longitudinal FA reduction in bvFTD patients compared to controls (Figure 2B). These differences were significant in the uncinate fascicle (bvFTD-stage 1, *p* < 0.004), in the corticostriatal pathway (bvFTD-stage 2, *p* < 0.008), and in the optic radiation (bvFTD-stage 4, *p* < 0.02); no significant differences were found in the CST (bvFTD-stage 3, *p* = 0.30) and in the reference tract (*p* = 0.51). The grand average, i.e., the average of FA-values in all stage-related tract systems, showed a significant FA-reduction for bvFTD patients vs. controls (*p* < 0.004). These longitudinal differences were calculated from FA maps after stereotaxic normalization with data harmonization to a time interval of 12 months (see Methods section “Data processing IV”).

The longitudinal tract-related differences in FA values showed no differences between bvFTD patients of different stages. No tendency to an accelerated or slowed alteration in a specific bvFTD-stage could be observed. However, a detailed subgroup analysis was not possible due to the limited sample sizes in the subgroups for the single stages.

### Tract-specific longitudinal FA decrease

**Figure 3:**
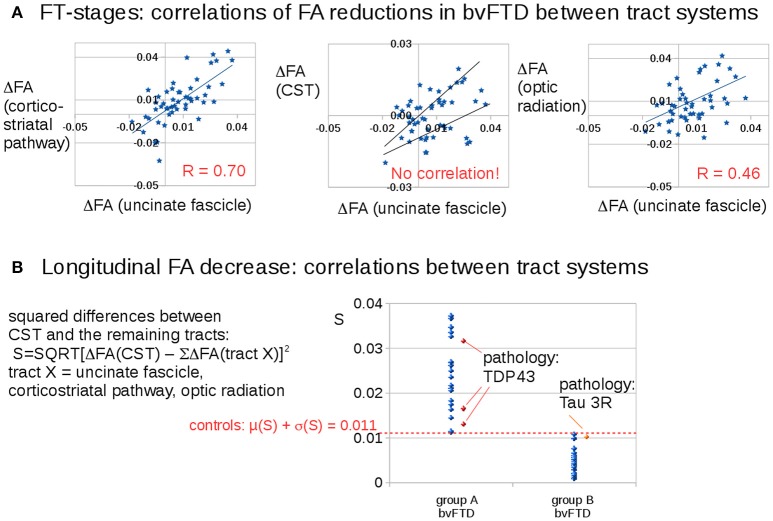
Longitudinal FA decrease in different tract systems: prominent differences for the CST (bvFTD stage 3). **(A)** Correlations of FA reductions in bvFTD patients between tract systems. **(B)** Squared differences of FA decrease in the CST compared to averaged FA decreases in the other tract systems. A “dichotomous” behavior was identified: one bvFTD subgroup A (*N* = 22) with an FA decrease in the CST which is different to the FA decrease in the other stage-related tract structures (S > 0.011) and the other subgroup (group B, *N* = 29) with an FA decrease in the CST which is similar to the FA decrease in the other stage-related tract structures (S < 0.011). The four available pathology results were indicated in red/orange.

Figure 3A shows that longitudinal FA alterations in the tract systems uncinate fascicle, corticostriatal pathway, and optic tract correlate with each other. This is not the case for the CST as the bvFTD stage 3-related tract system: longitudinal FA alterations in the CST could be subdivided into two groups with different correlations with the uncinate fascicle (as well as to the remaining tract systems). Then, the differences between longitudinal FA alterations in the CST and longitudinal FA alterations in the remaining tracts were calculated as follows,

(6) $$\begin{array}{l} \;S\;=SQRT{\left[\Delta FA(CST)-\Sigma \Delta FA(tractX)\right]}^{2}.\\ tract\;X\;=uncinate\;fascicle,corticostriatal\;pathway,\\ \;optic\;radiation \end{array}$$

Figure 3B shows a discrimination into two groups: Group B (*N* = 27) shows longitudinal FA alterations that are the same for all tract systems, whereas group A (*N* = 22) shows longitudinal FA alterations in the CST that are different from longitudinal FA alterations in the remaining tracts. The separation between the subgroups was defined by the control group which showed a variation of the parameter S of 0.011 (μ + σ). The differences in Figure 3B result from subjects in different stages.

Pathology results from four bvFTD-patients revealed 1 tau-mutation and 3 TDP43 mutations. The classification of these pathology cases according to Equation (1) showed the 3 TDP43-mutations in group A and the tau-mutation in group B.

### Whole brain-based spatial statistics—WBSS

**Figure 4:**
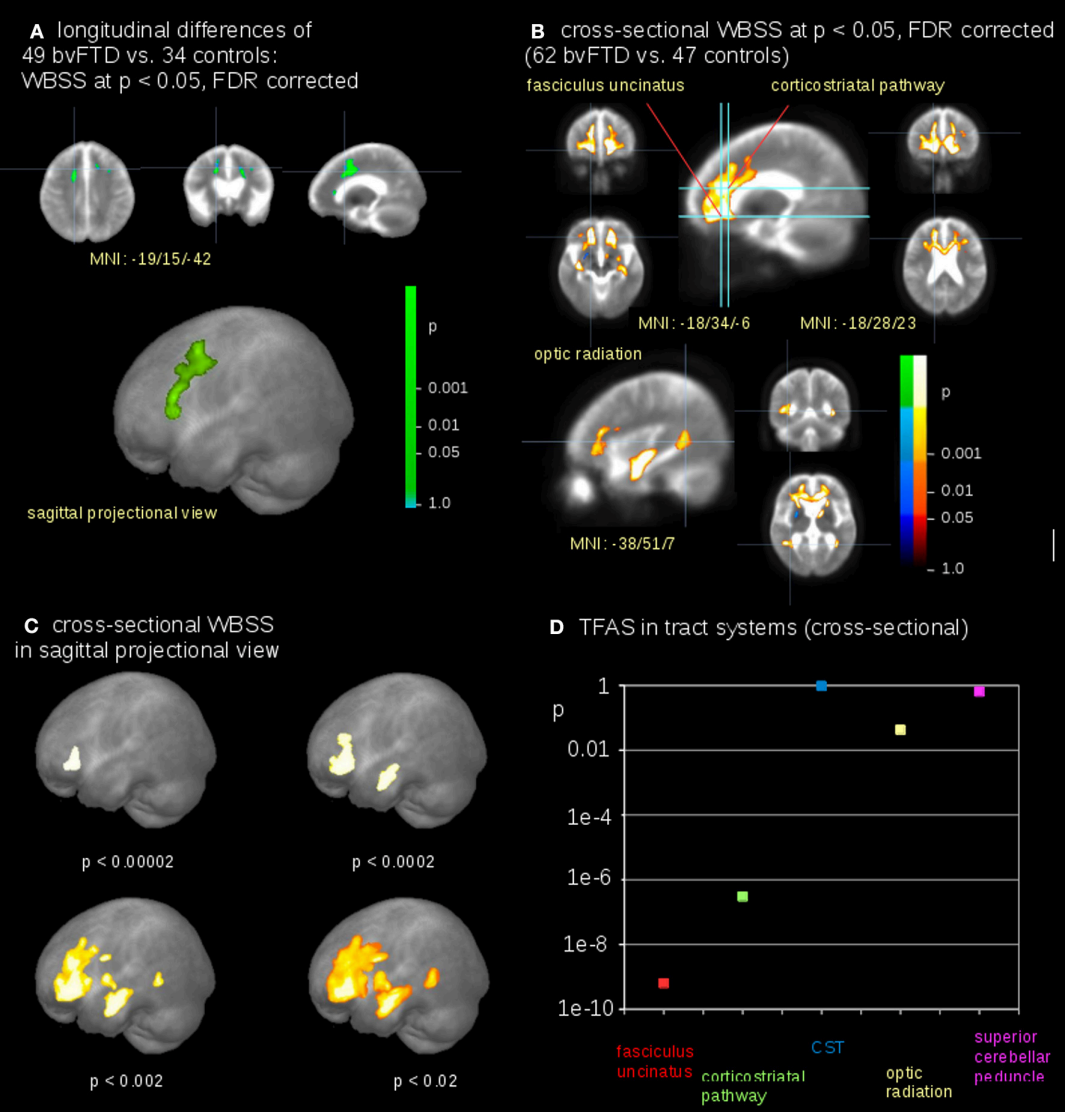
Comparison at the group level—bvFTD-patients vs. controls. **(A)** Whole brain-based spatial statistics (WBSS) of longitudinal alterations in FA maps of 49 bvFTD-patients vs. 34 controls (at FDR corrected *p* < 0.05). **(B)** WBSS of FA maps of 62 bvFTD-patients vs. 47 controls (at FDR corrected *p* < 0.05). **(C)** Whole brain-based spatial statistics (WBSS) of FA maps (FDR corrected) in sagittal projectional views for different significances. **(D)** Significance levels for TFAS of tract systems corresponding to bvFTD-stages: fasciculus uncinatus (bvFTD stage 1), corticostriatal pathway (bvFTD stage 2), corticospinal tract (CST) (bvFTD stage 3), optic radiation (bvFTD stage 4), and as the reference path the superior cerebellar peduncle.

The voxelwise comparison of FA differences between bvFTD patients and controls revealed significant differences in the frontal lobes (Figure 4A); FA-values were linearly corrected to the identical time interval of 12 months prior to the comparison at the group level (see Methods section “Data processing IV”). After correction for scanner differences and age, baseline scans of 62 bvFTD patients and 47 controls underwent WBSS. WBSS result patterns demonstrated extensive clusters mainly in the frontal lobes as well as in the optic radiation and posterior temporal white matter (Figures 4B,C). A summary of the resulting clusters is provided in Supplementary Table 1.

The highest significances in differences at the group level were found in the frontal lobes (*p* < 0.00002), followed by clusters reflecting hippocampal involvement (*p* < 0.0002 and *p* < 0.002, respectively), and then significances (*p* < 0.02) for alterations including the optic radiation, *p*-values were corrected for multiple comparisons. This significance cascade is illustrated in Figure 4C.

The TFAS-based analysis of tract systems showed a similar significance pattern compared to WBSS, with the highest differences of FA values between bvFTD patients and controls in the uncinate fascicle (*p* < 0.0000000001) and in the corticostriatal pathway (*p* < 0.0000004). In addition, the TFAS-based analysis was also significant in the optic radiation (*p* < 0.04), but not in the CST (*p* = 0.96) and in the reference tract (*p* = 0.64; Figure 4D).

### bvFTD staging by TFAS

**Figure 5:**
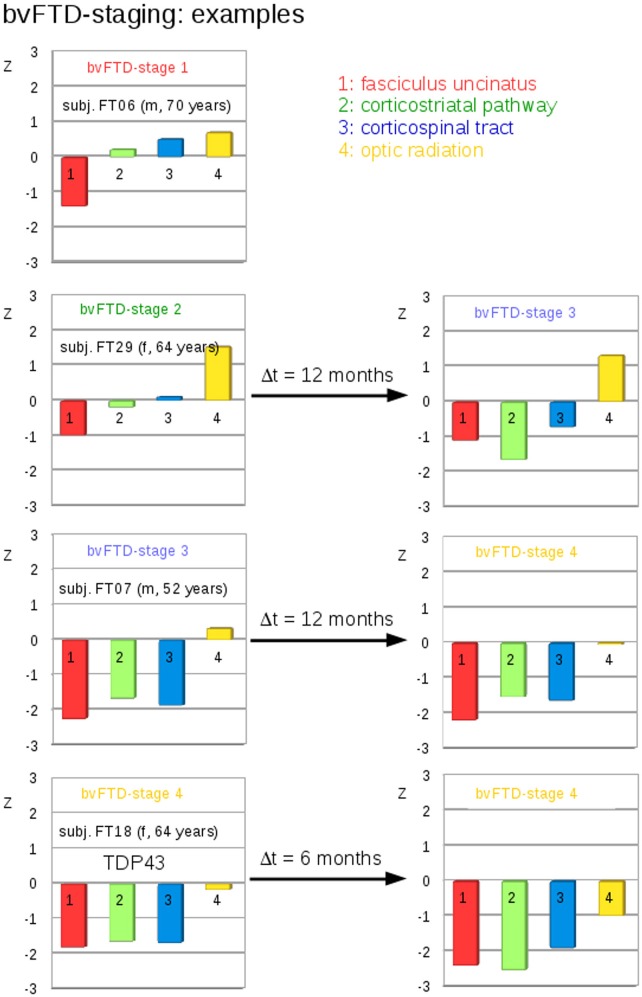
Individual examples for the staging categorization. Individual cross-sectional examples for the categorization of bvFTD patients into bvFTD stages based upon deviations of z-transformed FA-values (TFAS) from controls' values for different bvFTD stages. **Left:** baseline categorization, **Right:** categorization at follow-up.

A categorization into the staging scheme (“bvFTD stage 1” to “bvFTD stage 4”—Figure 1B; Methods section “Data processing VI”) was performed by use of the staging scheme (“bvFTD stage 1” to “bvFTD stage 4”) using the decision algorithm following Kassubek et al., 2014; the results are summarized in Supplementary Table 2. FA values of the uncinate fascicle of bvFTD patients showed a sensitivity of 81% in the separation from controls' FA values. This means that 81% of the baseline scans could be categorized. The staging categorization was performed independently for baseline and for follow-up data following the identical rules. Examples for patients in bvFTD-stages 1–4 are summarized in Figure 5, showing bvFTD-staging examples of baseline and follow-up scans, displaying the decrease in FA values in the respective tract systems. Fifty patients (81%) could be classified at the baseline scan. Classification was not possible in the remaining 12 since they showed no abnormalities in the uncinate fascicle (bvFTD stage 1 related tract). However, in the follow-up data sample, 42/49 bvFTD patients (86%) could be classified. Of these, seven patients showed an increase in the bvFTD stage (Table 3), 35 showed a constant stage, and seven were not classifiable. One bvFTD patient who had not been classifiable at baseline could be classified at the follow-up scan, five showed an increase of one stage, and one showed an increase of two stages. The tract-related differences in FA values were distributed over bvFTD stage 1 to bvFTD stage 4, no tract-related difference specific to single stage structures could be observed. A detailed subgroup analysis was not possible due to a low N in the subgroups for the respective stages. The differences in Figure 2B occur from subjects in different stages. At baseline and at follow-up, 8 bvFTD patients that were categorized as bvFTD stage 2 show alterations in tract 4 associated tracts (Table 3).

**Table 3:** Staging categorization.

**bvFTD-stage**	**At baseline (*N* = 62)**	**At follow-up (*N* = 49)**
1	10	4
2	24^*^	23^*^
3	9	8
4	7	7
not stageable	12	7
∑	62	49

* *At baseline and at follow-up, 8 out of 24 bvFTD patients already showed involvement of the optic radiation*.

### Comparison to neuropathology

Neuropathology results were available from four bvFTD-patients. They revealed one Tau-mutation and three TDP-43 mutations (Figure 3B). The classification of these cases according to Equation (1) showed the three TDP-43 mutations in group A, and the Tau-mutation in group B.

## Discussion

### *In vivo* imaging of TDP-43 pathology in bvFTD

After the development of a concept for patterns of TDP-43 pathology in bvFTD that showed a possible sequential progression of pTDP-43 aggregates (Brettschneider et al., 2014), the task remained to see if *in vivo* neuroimaging measures might be identified which were consistent with these proposed patterns of progression. Here, we adapted the DTI/TOI-based staging approach (Kassubek et al., 2014), which was developed for the proposed sequential progression of ALS neuropathology (Braak et al., 2013), to bvFTD.

### Cross sectional and longitudinal alteration patterns in bvFTD

The neuroimaging patterns observed in this study reflected the characteristic damage pattern of frontotemporal structures cross-sectionally as well as longitudinally. This “classical” finding has been included in the current definition of the diagnostic criteria, with one item (III.C.1.) for probable bvFTD reading as follows: “…imaging results consistent with bvFTD must be present: frontal and/or anterior temporal atrophy on MRI or CT” (Rascovsky et al., 2011). Beyond any reasonable doubt, this atrophy pattern represents the characteristic and macroscopically accessible structural imaging finding in bvFTD that can also be observed by functional radioligand neuroimaging, as also noted in the revised diagnostic criteria (III.C.2., “frontal and/or anterior temporal hypoperfusion or hypometabolism on PET or SPECT”; Rascovsky et al., 2011). There is ample evidence for these findings from a multitude of neuroimaging studies across modalities, as confirmed by a recent meta-analysis by use of the anatomical likelihood estimate method (Schroeter et al., 2014). In the current study, the WBSS analysis resembled this pattern. Remarkably, similar DTI changes in the posterior temporal WM have been reported in bvFTD cross-sectionally and longitudinally (Agosta et al., 2012; Lam et al., 2014; Mahoney et al., 2015).

### Tract definitions

With the exception of pattern 1, each of the tracts selected ends in the regions defined where TDP-43 pathology was reported: pattern 2: corticostriatal/accumbens nucleus; pattern 3: corticospinal tract/spinal cord ventral horn; pattern 4: optic radiation/primary visual cortex. Granted, for pattern 1 (uncinate fascicle/basal and anterior prefrontal neocortex), the uncinate fascicle extends further into anteromedial portions of the temporal lobe; but it is equally clear that, in bvFTD, neuropathology is present in temporal lobe structures with the exception of the superior temporal gyrus (The Lund and Manchester groups, 1994; Brettschneider et al., 2014). Importantly, it cannot be claimed that the results presented here confirm TDP-43-based staging, as other staging schemes/pathological proteins (e.g., tau, SOD1) were not tested, and it is not known whether these subjects were indeed TDP-43-positive subjects.

### Hypothesis guided tract-based analysis

Specifically, the TOI-based approach used in this study is a microstructural measure of correlates of the progressive pathological process. This technique targets distinct anatomical tract structures that correlate with the proposed progression pattern based upon histopathology (Brettschneider et al., 2013) and are not *per se* identifiable by mere inspection of MRI structure and not even by data-driven (perhaps whole-brain based) computerized analyses. The approach to analyze a “progression” pattern is longitudinal in nature. As such, the analysis according to the progression concept—which has been developed on the basis of cross-sectional postmortem data—aims at the identification of patterns that can be consistently found in a diverse group of neurodegenerative disorders, such as FTLD, each of which entails the aggregation of abnormal protein inclusions in characteristic patterns and locations (Jucker and Walker, 2013).

For the complete sample, the highest significance in discriminating between patients with bvFTD and controls was observed for the early tract patterns 1 and 2, and the significance level of the comparisons at the group level was lower the higher the disease stage at which the corresponding tracts were involved. The differences in the optic radiation (bvFTD stage 4-related tract) were significant, although a high variability in the controls sample was observed. In the longitudinal data with a time interval of about 12 months, three tract structures showed significant longitudinal FA decrease.

These results are in a general line of agreement with the recently proposed neuropathological bvFTD propagation pattern (Brettschneider et al., 2014). In the staging categorization, seven bvFTD patients showed a progression of bvFTD stage and 42 bvFTD patients showed a stable bvFTD stage, thus underlining the plausibility of the neuroimaging staging concept. This concept allows for a classification of about 80% of the patients (compare also Kassubek et al., 2014), and longitudinal assessment resulted in a lower number of unclassified cases.

## Limitations

The present study is not without limitations. A technical limitation of this study is the assumption that the alteration process in FA values is linear, with the consequence of a linear correction of FA-value differences to the identical time interval of 12 months prior to the comparison at the group level. This assumption was a first order approximation and does not incorporate that data at different clinical severity could cause changes in DTI measures that are not linear in time. However, given the lack of a uniform theory about the longitudinal alterations of FA (and other DTI metrics), FA alterations were approximated as a linear process in the observed average time range of about one year.

A further limitation of this method is that intrinsically (due to a sensitivity < 100%) only between 80 and 90% of the data could be categorized.

A major limitation of any neuroimaging approach, including the present one, is the lack of autopsy-confirmed data. Thus, our TOI-based analysis can provide only a plausible surrogate *in vivo* “staging” pattern for the presumed pathology in the bvFTD cohort studied. The findings require confirmation in longitudinal autopsy-controlled studies, i.e., to date only four cases have been confirmed neuropathologically. This number is insufficient to know with certainty whether the proposed approach works. Therefore, the results concerning the “dichotomous” behavior of CST-related FA changes are interpreted as possibilities rather than as validated results. In this context, one limitation derives from neuropathological observations that FTLD presenting as bvFTD showed pTDP-43 pathology in only about 45–50% of cases, whereas other cases are defined by neuronal inclusions of the microtubule-associated protein tau or, less frequently, by aggregates of the RNA-binding protein FUS (Ling et al., 2013). To date, there is no *in vivo* test that makes it possible to reliably differentiate FTLD-TDP from FTLD-tau or—FUS, and the cohort in the present study most likely included cases with mixed underlying protein pathologies, for which scant autopsy data were available. Therefore, sequential fiber tract involvement, as observed here, is likely to reflect progression of disease pathology in bvFTD cases in general, although our results may or may not be specific to cases with pTDP-43 pathology. In a recent autopsy study of 21 individuals with Pick's disease (PiD), tau lesions (FTDL-tau) were detected in some of the same regions as the pTDP-43 pathology in pTDP-43 bvFTD cases but not always at the same phases, e.g., orbital gyri, superior and middle temporal gyri (PiD phase 1, one case), middle frontal and anterior cingulate gyri, striatum, primary somatosensory neocortex (PiD phase 2, three cases), agranular motor neocortex, precerebellar nuclei of the brainstem (PiD phase 3), primary visual neocortex (PiD phase 4) (Irwin et al., 2016).

The previous publication on which the present neuroimaging study is based referred to “patterns” rather than “stages” of TDP-43 pathology in bvFTLD, i.e., it was not intended to serve as a neuropathological staging system for all FTLD-TDP and it excluded cases with PiD (Brettschneider et al., 2014). The *N* = 39 cases with bvFTD examined by Brettschneider et al., 2014 had varied genetic backgrounds and differed with respect to their clinical phenotypes; nevertheless, they displayed similar patterns of TDP-43 pathology in the end phase of the disease at autopsy (Brettschneider et al., 2014). Within the pTDP-43-associated variant itself, a differentiation between the dendritic/neuritic (nFTLD) and pericaryal/cytoplasmic (cFTLD) types on clinical grounds is still not possible (Brettschneider et al., 2014). Whereas, cases with nFTLD pathology were reported to show widespread and severe neocortical involvement from orbitofrontal areas along the entire convexity of the brain that frequently reached the occipital cortex, cFTLD cases by contrast showed less severe neocortical involvement (Brettschneider et al., 2014). Thus, it is difficult to speculate here about the possible impact of the cFTLD/nFTLD proportion of our sample on the DTI results. However, to the extent that none of the cases in the present cohort showed deficits associated with motor neuron disease, and inasmuch as the cFTLD type pathology appears to be more closely related to ALS than the nFTLD type—cFTLD cases are not only much more likely to have clinical signs of motor neuron disease (*p* < 0.001, Brettschneider et al., 2014) but also a shorter survival period—the lack of involvement on the part of the CST might not be not entirely surprising.

A potential approach to this open question might be derived from the longitudinal data of this study: The “dichotomous” behavior of CST-related FA changes could be interpreted as an indicator for the presence of pTDP-43 pathology of the cFTLD type in the sample with an FA decrease in CST that is different from the FA decrease in the other stage-related tract structures. This interpretation that the CST may differentiate between these neuropathological subtypes, however, remains speculative and is data-driven by the “dichotomous” results in the CST. As such, it will require further confirmation by autopsy studies or needs to be supported by more longitudinal data sets.

The mean disease duration differed between the three sites. As a consequence the longitudinal alterations were calculated as differences between two scans so that they were independent of the scanner type. In that respect, differences in average disease duration between centers did not affect the analysis and the bvFTD group can be considered as one group with an average disease duration of 5 ± 4 years. However, no correlation of the staging with clinical/behavioral severity measures could be found. Behavioral severity measures were not available for many data sets; thus, this correlation could not be calculated.

### Hyperphosphorylated tau pathology

According to a recent autopsy-based study, hyperphosphorylated tau pathology was present in 98% of the 296 cognitively unimpaired subjects ranging from age 50 to 102 years of age; Aβ was seen in 50%, TDP-43 in 36%, and α-synuclein in 19% of the subjects (Elobeid et al., 2016). Thus, it is not unreasonable to assume that the individuals in the present cohort could have mixed pathologies. On the other hand, it is also true that most cases with autopsy-confirmed bvFTD do not display high AD-associated NFT and Aβ stages or α-synuclein Parkinson's disease-related stages (Brettschneider et al., 2014; Perry et al., 2017). Moreover, although our cases with bvFTD may have varied genetic backgrounds, Brettschneider et al., 2014 showed that even such individuals revealed similar patterns of TDP-43 pathology at end stage disease upon postmortem examination. Even where somewhat different white matter profiles were seen to be associated with genetic sub-groups of bvFTD (e.g., MAPT and C9ORF72 mutations, the main point is that bvFTD can be distinguished from AD (Mahoney et al., 2014). As such, we do not believe the presence of concurrent pathological proteins in our cohort would have had a major impact on the present neuroimaging findings.

### Summary

In summary, the hypothesis-driven analysis of the neuropathologically-defined progression pattern with the *in vivo* TOI-based technique was applied to the bvFTD sample and suggested a characteristic pattern of damage to the involved major white matter pathways both cross-sectionally and longitudinally.

It has already been demonstrated that progressive regional accumulation of pTDP-43 inclusions in ALS reflects sequential involvement of white matter fiber tracts of the central nervous system (Kassubek et al., 2014, 2017; Müller et al., 2016). While no direct neuroimaging marker for pTDP-43 exists at present, several earlier neuropathological studies have emphasized the close correlation between the severity of pTDP-43 aggregation and neuro-axonal loss (Geser et al., 2008, 2009). Therefore, this study is interpreted as a first attempt to apply DTI—which has already successfully proven to serve as a marker for microstructural integrity in pTDP-43 related neurodegeneration in ALS (Kassubek et al., 2017)—as a possible marker for microstructural integrity to bvFTD. The DTI investigation of microstructural integrity is a distinct technique to regional volumetric analysis which measures directly regional atrophy and, in that manner, could serve as a complementary neuroimaging marker.

This approach might have the potential for future use in the work-up of individual patients, thereby also potentially enlarging the spectrum of non-invasive markers as a neuroimaging-based read-out for clinical studies and supporting the identification of patients who might be eligible for trials targeted at treating the specific histopathologic abnormalities causing FTLD (Grossman, 2010).

## Author contributions

JK and H-PM: study concept and design, data analysis, and interpretation of data, critical revision of manuscript for intellectual content; KD, MG, HB, and AL: interpretation of data, critical revision of manuscript for intellectual content; MH: data collection, interpretation of data, critical revision of manuscript for intellectual content; MS, KM, SA-S, IU, JH, OP, and MO: data collection, critical revision of manuscript for intellectual content.

### Conflict of interest statement

The authors declare that the research was conducted in the absence of any commercial or financial relationships that could be construed as a potential conflict of interest.
